# Structural definition of hSP-D recognition of *Salmonella enterica* LPS inner core oligosaccharides reveals alternative binding modes for the same LPS

**DOI:** 10.1371/journal.pone.0199175

**Published:** 2018-06-18

**Authors:** Jamie R. Littlejohn, Ruben F. da Silva, William A. Neale, Carrie C. Smallcombe, Howard W. Clark, Rose-Marie A. Mackay, Alastair S. Watson, Jens Madsen, Derek W. Hood, Ian Burns, Trevor J. Greenhough, Annette K. Shrive

**Affiliations:** 1 School of Life Sciences, Keele University, Staffordshire, United Kingdom; 2 University of Southampton, Department of Child Health, Division of Clinical and Experimental Sciences, Sir Henry Wellcome Laboratories, Southampton General Hospital, Southampton, United Kingdom; 3 Southampton NIHR Respiratory Biomedical Research Unit, Southampton General Hospital, Southampton, United Kingdom; 4 Institute for Life Sciences, University of Southampton, Southampton, United Kingdom; 5 Mammalian Genetics Unit, MRC Harwell Institute, Harwell Science and Innovation Campus, Oxfordshire, United Kingdom; Nanyang Technological University, SINGAPORE

## Abstract

The crystal structures of a biologically and therapeutically active recombinant homotrimeric fragment of native human SP-D (hSP-D) complexed with the inner core oligosaccharide of the *Salmonella enterica* sv Minnesota rough strains R5 and R7 (rough mutant chemotypes Rc and Rd1) have been determined. The structures reveal that hSP-D specifically and preferentially targets the LPS inner core via the innermost conserved Hep-Kdo pair with the flexibility for alternative recognition when this preferred epitope is not available for binding. Hep-Kdo binding is achieved through calcium dependent recognition of the heptose dihydroxyethyl side chain coupled with specific interactions between the Kdo and the binding site flanking residues Arg343 and Asp325 with evidence for an extended binding site for LPS inner cores containing multiple Kdo residues. In one subunit of the R5-bound structure this preferred mode of binding is precluded by the crystal lattice and oligosaccharide is bound through the terminal inner core glucose. The structures presented here thus provide unique multiple insights into the recognition and binding of bacterial LPS by hSP-D. Not only is it demonstrated that hSP-D targets the highly conserved LPS proximal inner core Hep-Kdo motif, but also that hSP-D can recognise either terminal or non-terminal sugars and has the flexibility and versatility to adopt alternative strategies for bacterial recognition, utilising alternative LPS epitopes when the preferred inner core Hep-Kdo disaccharide is not available for binding.

## Introduction

The innate immune protein human surfactant protein D (hSP-D) is a collagenous C-type lectin prototypically associated with the lung surfactant but now identified throughout the human body [1]. SP-D recognises a wide range of pathogens, through their surface carbohydrate arrays, leading to rapid deactivation and clearance through activation of phagocytic cells [2–4].

Numerous studies of the binding of simple sugars to both hSP-D and rfhSP-D, a recombinant homotrimeric native fragment comprised of the neck region plus three carbohydrate recognition domains (CRDs) which exhibits significant biological and therapeutic activity *in vivo* and *in vitro* [5–10] have been carried out both in solution and through 3-dimensional structural studies. Binding studies which demonstrate affinity for simple sugars such as glucose, mannose, maltose, heptose and inositol [11–13] have been confirmed crystallographically (see for example [14–18]). In all cases recognition is through CRD Ca-dependent binding of the terminal monosaccharide through a mannose-type equatorial hydroxyl pair O3' and O4' or a stereochemically equivalent pair.

A number of studies of gram-negative bacteria, including *Escherichia coli*, *Salmonella enterica* serovar Minnesota and *Haemophilus influenzae* [19,20] have reported that lipopolysaccharide (LPS) on the bacterial surface mediates hSP-D binding with LPS recognition initially thought to be via terminal carbohydrate residues through carbohydrate recognition domain Ca-dependent binding of a mannose-type equatorial hydroxyl pair on the terminal monosaccharide [14–16]. Our recent structural definition of the recognition of *H*. *influenzae* Eagan strains inner core LPS by a recombinant fragment of native hSP-D (rfhSP-D) [6,7,21] revealed that recognition occurs by calcium-dependent binding of the non-terminal heptose in the proximal inner core, with the Kdo positioned to interact with the binding site flanking residues Asp325 and Arg343 [15,16]. Furthermore, a recent study by Reinhardt and colleagues has identified that a number of synthetic oligosaccharide cores can be bound by wildtype hSP-D with increased affinities for heptose-rich cores and a clear preference for a terminal Hep-Kdo motif compared to Hep alone [22].

*Salmonella enterica* is a gram-negative bacterium that causes widespread outbreaks of gastroenteritis and bacteraemia in humans, with the most severe *S*. *enterica* Typhi infections causing enteric fever (reviewed by Coburn *et al*. [23]). Serovar Minnesota has been identified as the causative agent in a number of gastroenteritis outbreaks and, more recently, urosepsis in a Crohn’s patient [24–26]. The LPS oligosaccharide can be divided into the core oligosaccharides, containing 3-deoxy-D-*manno*-oct-2-ulosonic acid (Kdo), L-*glycero*-D-*manno*-heptose (Hep), α-D-glucose (Glc), α-D-galactose (Gal) and N-*acetyl*-D-glucosamine (GlcNAc), and the antigen-specific O-oligosaccharides that extend in repeats away from the bacterial cell surface [27]. In *Salmonella* species the inner and, to a large extent, the outer core oligosaccharides are a conserved epitope [28].

**Fig 1:**
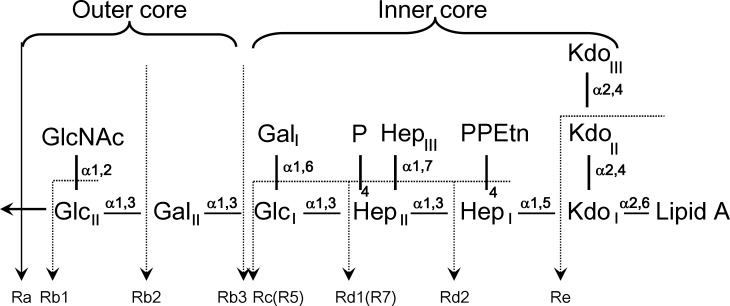
Core LPS structure of *Salmonella enterica* rough mutant strains. The LPS phenotypes (Ra, Rb1, Rb2, Rb3, Rc, Rd1, Rd2, and Re) and the mutant strains R5 and R7 are shown (modified from Mansfield and Forsythe, 2001 [29]). Glc: glucose; GlcNAc: N-acetyl-glucosamine; Gal: galactose; Hep: L-D-Heptose; P: phosphate; PEtn: phosphoethanolamine; Kdo: 3-deoxy-D-*manno*-oct-2-ulosonic acid.

Rough mutants of *S*. *enterica* Minnesota lack the O-antigen, leaving the core sugar residues exposed to the immune system leading to the possibility of recognition by SP-D in a similar manner to that reported for *H*. *influenzae* Eagan 4A [21]. While the LPS core is largely conserved, mutations in the synthetic enzymes responsible for assembling the LPS can result in truncated oligosaccharide cores such as those of strains R5 and R7, equivalent to the Rc and Rd1 phenotypes (Fig 1), demonstrated through lectin blotting and agglutination-inhibition studies to be recognised by hSP-D [19]. The R5 and R7 rough strain LPS exhibit similar structures with the R5 extended by an extra glucose (GlcI) bound to HepII. The R5 strain arises from a single mutation in the phosphorylation step of HepI, inhibiting and impacting on the phosphorylation of HepII and the addition of GalI and GalII to GlcI. Phosphorylation of HepI and HepII initiates the extension of GalI and GalII from GlcI, producing the Rb2 strain [30–33].

While the *H*. *influenzae* Eagan 4A LPS [21] includes a truncated inner core with a single Kdo and a single Hep with a single Glc extension, the *S*. *enterica* Minnesota strains R5 and R7provide the opportunity to investigate, through crystal structure analysis, host recognition of more complex natural LPS structures with inner cores containing multiple Kdo and Hep alongside a terminal Glc in the R5 strain. Here we show that hSP-D recognition of *Salmonella enterica* sv Minnesota rough strains R5 and R7 (rough mutant chemotypes Rc and Rd1) LPS occurs preferentially through the inner core Hep-Kdo, with hSP-D having the flexibility and versatility to target the terminal glucose of R5 when the preferred epitope is not accessible for binding.

## Materials and methods

### *S*. *enterica* Minnesota R5 and R7 oligosaccharide preparation

The oligosaccharide (delipidated) portions of *Salmonella enterica* sv. Minnesota R5 (Enzo Lifesciences-ALX-581-017-L002) and R7 (Enzo Lifesciences-ALX-581-018-L002) mutants were prepared under mild hydrolysis conditions with 2% acetic acid, in accordance with the method previously published by Masoud *et al*. [34] and our previously published procedures [21]. For the R5 LPS the molecular weight was calculated as ≈ 3036 Da by the addition of all glycoconjugates and Lipid A. The lipid A portion accounted for ≈ 1800 Da [35], making up 60% of the LPS. 2 mg of R5 LPS were initially hydrolysed, yielding ≈0.7 mg of the oligosaccharide representing 88% hydrolysis efficiency. The R5 oligosaccharide was dissolved in deionised water to give a KdoI-HepI-HepII-GlcI concentration of ≈16 mM (accounting for the beta elimination of KdoII-KdoII) for storage and for use in crystal soaking. The same procedure was followed for the R7 LPS providing a stock solution of ≈18mM for storage and for use in crystal soaking and cryoprotection preparation.

### Generation and purification of a recombinant fragment of hSP-D

A recombinant homotrimeric fragment of human SP-D (rfhSP-D) was expressed and purified as described previously [5,6,14]. Each chain contains 177 amino acids (Gly179 to Phe355) comprising a short collagen region with eight Gly-X-Y repeats, the α-helical coiled coil neck region and the globular CRD. The contaminating level of endotoxin present in the rfhSP-D preparation was minimized [6] using a Polymyxin B column (Detoxi-Gel, Pierce, UK) to remove endotoxin followed by assaying with the QCL-1000 Limulus amoebocyte lysate system (BioWhittaker, Walkersville, MD) according to manufacturer's instructions. The assay was linear over a range of 0.1–1.0 EU/ml (10 EU = 1 ng of endotoxin).

### Crystallisation, ligand soaking and cryoprotection

The endotoxin treated rfhSP-D at 8.8 mg/ml in PBS was used for crystallisation both directly (for the R7 studies), following addition of CaCl_2_ to give 10mM, and after dialysis (for the R5 studies) into 50 mM tris(hydroxymethyl)aminomethane (Tris), 10 mM CaCl_2_, 150 mM NaCl, 0.02% NaN_3_ at pH7.4. Protein concentrations used were 7.7–8.9 mg/ml. Native crystals of rfhSP-D were grown in sitting drops using screens around conditions previously reported [14]. Those for soaking with the delipidated R5 LPS were grown in precipitant buffer 0.1 M tris pH 8.0, 16% polyethylene glycol (PEG) 6000, and for the delipidated R7 LPS in 0.1 M Tris pH 7.0, 16% PEG 10000. Cryoprotectant buffers were prepared as previously described [21] to provide increasing concentrations of MPD (2,4-methylpentane diol) in precipitant buffer with ligand added to provide final concentrations of ≈8 mM and ≈13 mM in the crystallisation drop for R5 and R7 respectively. The crystals were soaked with ligand and prepared for cryocooling by successive addition of cryoprotectant containing increasing concentrations of MPD (5–15% for R7; 5–20% for R5) in 2 μl aliquots over three minutes followed by a final exchange of 6–8 μl of the drop with 6–8 μl of the final MPD cryoprotectant solution (15% MPD for R7; 20% MPD for R5). Crystals were flash-frozen at 100 K after a total of 24 minutes (R5) or 8 minutes (R7) soaking with ligand and cryoprotectant buffer.

### Data collection, structure solution and refinement

Data for the rfhSP-D R5 and R7 complexes were collected at 100 K at Diamond Light Source, Oxfordshire, on beamlines I04-1 and I03, respectively, using Dectris Pilatus 6M hybrid photon counting detectors. Integrated intensities were calculated using the program *MOSFLM* [36]. The 1.6 Å native structure of rfhSP-D (PDB ID 1PW9 [14]) was used as a starting model for modelling both the rfhSP-D/R5 and rfhSP-D/R7 structures. Electron density maps were calculated using the CCP4 suite of programs including *AIMLESS*, *TRUNCATE*, *SORTMTZ* and, where necessary, *REINDEX* [37]. Model building was completed using *COOT* [38] and refined using maximum likelihood refinement in *REFMAC5* [39], available as part of the CCP4 package, in alternating rounds. Ligand topology and parameter files were generated using *ProDRG* [40] following fitting into the density. Final refinement statistics are presented in Table 1 and the quality of the final model was confirmed using the MolProbity web server [41]. The coordinates and structure factors for the R5-bound (PDB ID 5OXS) and R7-bound (PDB ID 5OXR) structures have been deposited with the Protein Data Bank. All main and side-chain stereochemical parameters are within accepted limits or better, with more than 98% of residues in favoured regions of the Ramachandran plot with no outliers. Molecular figures were generated using CCP4mg molecular graphics software [42] and the PyMOL Molecular Graphics System Version 1.4 (Schrödinger, LLC, 2011).

**Table 1:** Data collection and processing.

*Data collection*	rfhSP-D/R7	rfhSP-D/R5
Synchrotron station	DLS I03	DLS I04-1
Wavelength (Å)	0.97625	0.92819
Space group	P2_1_	P2_1_
Cell dimensions	a = 55.33 Å, b = 108.14 Å, c = 55.67 Å, β = 91.82⁰	a = 55.68 Å, b = 108.51 Å, c = 56.17 Å, β = 92.89⁰
Resolution range (Å)	55.3–1.75 (1.78–1.75)	56.10–1.65 (1.68–1.65)
Observations	143,142 (7,653)	230,306 (10,955)
Unique reflections	61,250 (3,299)	74,199 (3,629)
Completeness (%)	93.2 (91.2)	93.0 (93.2)
Rmerge ^a^	0.066 (0.359)	0.054 (0.397)
Mean (I/σ(I))	7.3 (2.2)	11.4 (2.5)
***Refinement***		
Protein atoms	3483	3537
Residues chain A	205–355	204–355
Residues chain B	204–355	203–355
Residues chain C	205–355	203–355
Water molecules	479	477
Other molecules		
Subunit	A B C	A B C
Calcium ions	3 3 3	2 2 1
Oligosaccharide	- 1 1	1 1 1
R_work_ ^b^ (%)	17.3	16.5
R_free_ ^c^ (%)	19.6	18.7
r.m.s.d. bond length (Å)	0.010	0.010
r.m.s.d. bond angle (°)	1.40	1.32
Average B-values (Å^2^)		
Protein	23.1	22.6
Water	34.2	34.9
Other hetero-atoms	31.1	40.2
PDB ID	5OXR	5OXS
***Ramachandran plot values*** *^d^* ***(%)***		
Favoured	97.8	97.8
Outliers	0.0	0.0

Figures in parentheses refer to the highest resolution bin

^a^ R_merge_ = ∑_h_∑_j_ |I_h,j_—I_h_| /∑_h_∑_j_ |I_h,j_|, where I_h_,_j_ is the j^th^ observation of reflection h and I_h_ is the mean of the j measurements of reflection h.

^b^ R_work_ = ∑_h_ ||F_oh_|—|F_ch_|| /∑_h_ |F_oh_| where F_oh_ and F_ch_ are the observed and calculated structure factor amplitudes, respectively, for the reflection h.

^c^ R_free_ is equivalent to R_work_ for a randomly selected subset (5%) of reflections not used in the refinement.

^d^ Defined according to Molprobity

## Results

The trimeric rfhSP-D structures comprising three carbohydrate recognition domains (CRDs) and the α-helical coiled-coil neck region have been refined to 1.65 Å and 1.75 Å resolution for the R5-bound and R7-bound structures respectively. In all but one of the CRDs the structures reveal oligosaccharide ligand in the Ca1 binding pocket. No ligand is present in subunit A of the R7-bound structure while the mode of oligosaccharide recognition in subunit A of the R5-bound structure differs from the Hep-Kdo binding seen in subunits B and C of both structures.

**Fig 2:**
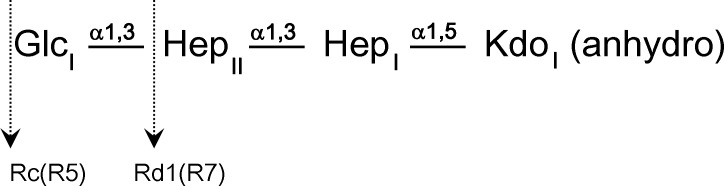
Structure of the product of mild acid hydrolysis of *S*. *enterica* Minnesota rough mutants R5 and R7. Glc: glucose; Hep: L-D-Heptose; Kdo: 3-deoxy-D-manno-oct-2-ulosonic acid.

**Fig 3:**
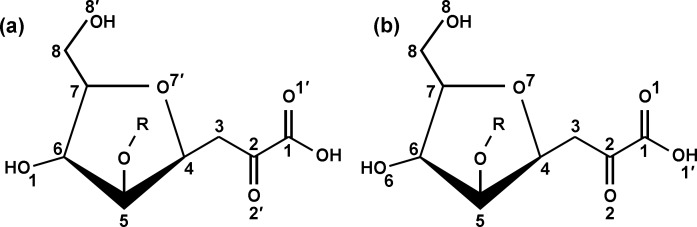
Structure of the 4,7 closure furanoid derivative (anhydro Kdo) of Kdo following LPS delipidation by mild acid hydrolysis and β-elimination of the Kdo O4 substituent, reported by Shrive and co-workers (PDB ID 4E52, ligand KD5). Anhydro Kdo is proposed to be a racemic mixture (Auzanneau *et al*., 1991 [43]) with the 2-oxobutanoic acid side chain at C4 both alpha and beta to the glycosidic bond (at C5). (a) Original Kdo numbering retained. (b) Numbering according to pdb entry 4E52 (alpha) and used for the structure here.

**Fig 4:**
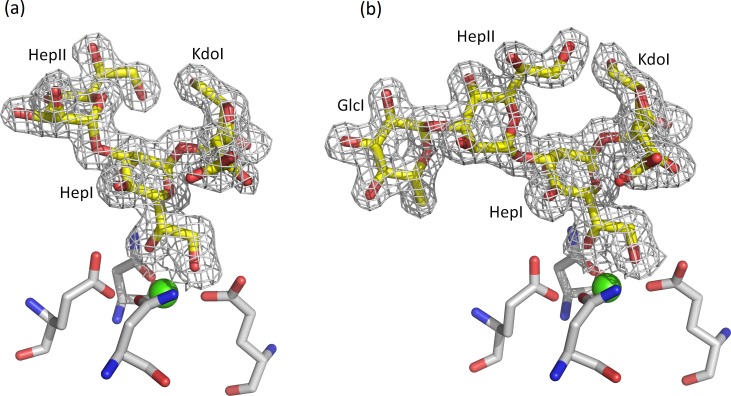
Electron density for the *Salmonella enterica* oligosaccharides bound to rfhSP-D (subunit B) with HepI in the Ca1 binding site. The calcium ion is in green and the four calcium and ligand coordinating residues Glu321, Asn323, Glu329 and Asn341 are shown in cpk. (a) R7 oligosaccharide KdoI (anhydro)-HepI-HepII (b) R5 oligosaccharide KdoI (anhydro)-HepI-HepII-Glc1. Maps are 2mFo-DFc contoured at 1σ.

The bound oligosaccharide is clearly defined in the electron density of both structures; Kdo-HepI-HepII for the R7 and Kdo-HepI-HepII-Glc1 for R5 (Fig 2). The Kdo is in the anhydro form (Fig 3) reported by Shrive and co-workers [21] in the rfhSP-D–*H*. *influenzae* Eagan 4A structure (pdb entry 4E52) but here the 5-membered ring and all substituents are fully defined in the electron density (Fig 4) except in subunit A of the R5-bound structure where LPS binding is not via HepI and the terminal anhydro Kdo is poorly defined. The Kdo electron density clearly shows the 2-oxobutanoic acid sidechain off C4 (Fig 3B) in a beta conformation with respect to the glycosidic bond in both structures. KdoII and KdoIII (Fig 1) are not present in the electron density.

**Fig 5:**
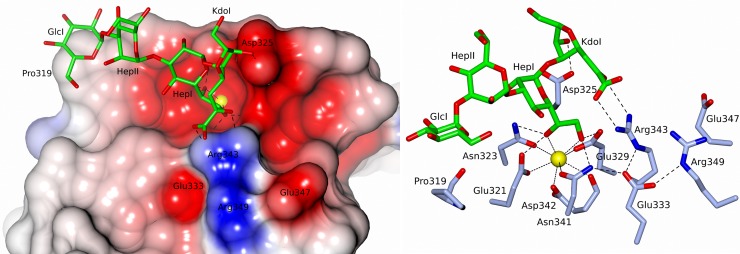
*S*. *enterica* R5 oligosaccharide bound to rfhSP-D (subunit C) with the inner core HepI in the Ca1 binding site. KdoI (anhydro) interacts with both Asp325 and Arg343. Similar binding is observed in subunit B and in subunits B and C of the R7-bound structure which lacks the terminal glucose GlcI. Calcium coordinating bonds are represented by dots and protein-ligand interactions by dashes. The interactions of Glu333 are also indicated.

In subunits B and C, in both structures, recognition of the oligosaccharide by rfhSP-D (Fig 5 and Table 2) is primarily via protein and Ca1 coordination of the 6'OH and 7'OH of the dihydroxyethyl side chain of HepI. Further HepI interactions with the binding pocket in these four subunits include those routinely associated with carbohydrate recognition by hSP-D [21].

**Table 2:** Calcium and ligand binding distances (Å).

Atom 1		Atom 2			R5			R7		
					A	B	C	A	B	C
Ca1		Glu	321	OE1	2.63	2.61	2.57	2.65	2.60	2.64
		Asn	323	OD1	2.46	2.42	2.47	2.49	2.44	2.38
		Glu	329	OE1	2.42	2.49	2.44	2.51	2.41	2.34
		Asn	341	OD1	2.50	2.44	2.44	2.37	2.43	2.44
		Asp	342	OD1	2.47	2.38	2.40	2.35	2.34	2.28
		Asp	342	O	2.61	2.55	2.62	2.54	2.51	2.51
		HepI		O6'	-	2.34	2.34	-	2.36	2.37
				O7'	-	2.38	2.34	-	2.35	2.34
		GlcI		O3'	2.38	-	-	-	-	-
				O4'	2.36	-	-	-	-	-
HepI	O6'	Glu	321	OE2	-	2.67	2.61	-	2.63	2.52
		Asn	323	ND2	-	3.01	2.92	-	3.03	2.93
	O7'	Glu	329	OE2	-	2.66	2.65	-	2.64	2.57
		Asn	341	ND2	-	2.98	3.04	-	2.94	3.07
GlcI	O3'	Glu	321	OE2	2.67	-	-	-	-	-
		Asn	323	ND2	2.76	-	-	-	-	-
	O4'	Glu	329	OE2	2.69	-	-	-	-	-
		Asn	341	ND2	3.00	-	-	-	-	-
KdoI	O1	Arg	343	NH2	-	3.09	2.87	-	-	-
KdoI	O2	Arg	343	NH1	-	3.09	3.26	-	3.10	3.06
KdoI	O6	Asp	325	OD2	-	2.80	2.56	-	2.76	2.69
GlcI	O6'	Arg	343	NH1	3.30	-	-	-	-	-
GlcI	O6'	Arg	343	NH2	3.22	-	-	-	-	-
HepI	O4'	Arg	343	NH2	3.06	-	-	-	-	-

**Fig 6:**
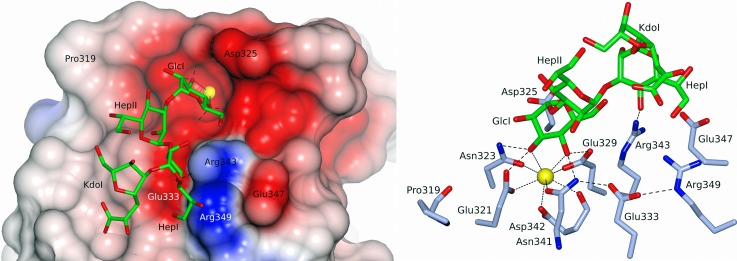
*S*. *enterica* R5 oligosaccharide bound to rfhSP-D (subunit A) with the terminal glucose GlcI in the Ca1 binding site. HepI interacts with Arg343. No ligand was observed in subunit A of the R7-bound structure due to crystal lattice constraints. Calcium coordinating bonds are represented by dots and protein-ligand interactions by dashes. The interactions of Glu333 are also indicated.

**Fig 7:**
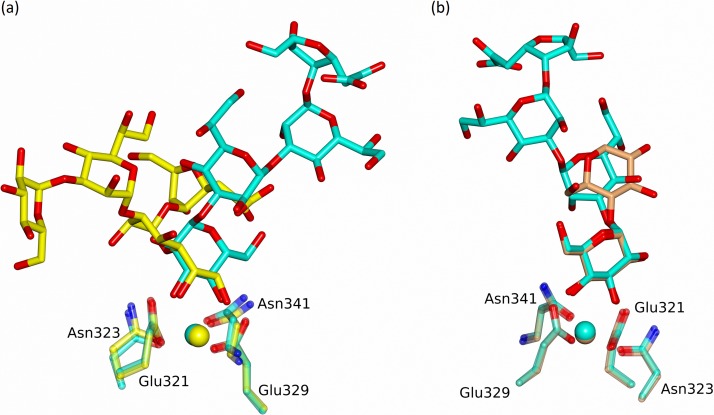
(a) Superposition of subunit A (GlcI bound) in cyan and subunit B (HepI bound) in yellow of the R5 LPS-bound structure following a least-squares fit of the CRD protein main chain atoms. (b) Superposition of subunit A (GlcI bound) in cyan of the R5 LPS-bound structure and subunit A (Glc1 bound) in coral of the maltose-bound structure [14] following a least-squares fit of the CRD protein main chain atoms.

In contrast to the Ca1-HepI binding in the B and C subunits of both structures, subunit A of the R5-bound structure reveals an alternative mode of LPS recognition with calcium-dependent binding of the terminal glucose GlcI of R5, with the remaining R5 oligosaccharide chain HepII-HepI-Kdo extending over Arg343 and the SP-A (surfactant protein A), SP-D conserved Arg349 and Glu333 (Fig 6). The relative positions of the LPS for the two alternative binding modes are shown in Fig 7A where least-squares fitting of the main chain CRD in the two cases reveals the completely different LPS orientations despite the conserved Ca1-OH interactions. The position of the Ca1-bound glucose GlcI and its interactions with Ca1 and the binding pocket closely mirror those reported in a variety of structures including the rfhSP-D maltose bound structure [14] as shown in Fig 7B. The Ca1 coordinates to the terminal glucose GlcI through 3'OH (2.38 Å) and 4'OH (2.36 Å). Protein coordination to both calcium (via Glu321, Asn323, Glu329, Asn341) and the calcium-bound Glc1 hydroxyls (via Glu321, Asn323, Glu329, Asn341, Asp342) follows the established pattern for ligand-bound SP-D structures (Table 2). Calcium Ca3, the more weakly coordinated of the three calcium ions Ca1, Ca2, Ca3 [15], is absent throughout the R5-bound structure. Ca2 is only weakly defined in subunit B of the R5-bound structure and is absent in subunit C where the calcium (Ca2 and Ca3) coordinating loop between Asp297 and Tyr304 is rearranged and a water molecule replaces Ca2.

In both structures and for both modes of binding (Ca1 coordination of HepI or GlcI) additional direct interactions between the protein and bound oligosaccharide are dominated by the binding-pocket flanking residues Arg343 and Asp325 (Table 2). For both ligands where HepI is coordinated to Ca1 (subunits B and C, both structures) the Kdo (anhydro) interacts with both binding site flanking residues, forming hydrogen bonds with Asp325 through Kdo O6 (2.56–2.80 Å) and with Arg343 through the extended 2-oxobutanoic acid sidechain of the anhydro Kdo (Fig 5 and Table 2). In subunit A of the R5-bound structure where GlcI is coordinated to Ca1, additional direct interaction between protein and oligosaccharide again involves Arg343 (Fig 6) which forms weak hydrogen bonds to both GlcI 6'OH and O4' of HepI (Table 2). The direct interactions between protein and oligosaccharide ligand in both structures and all subunits where oligosaccharide ligand is present are supplemented by extensive water molecule networks linking the ligand to the protein.

## Discussion

The structures of rfhSP-D, a biologically active recombinant native fragment of hSP-D, bound to inner core structures of *S*. *enterica* Minnesota LPS from deep rough mutant strains reveal SP-D targeting of the proximal inner core Hep-Kdo motif through calcium-dependent binding of the (non-terminal) Hep supplemented by interaction of both Hep and Kdo with the binding site flanking residues Arg343 and Asp325. The structures also provide a definitive demonstration for the first time that hSP-D has the versatility to recognise alternative LPS epitopes when the Hep-Kdo inner core unit is inaccessible. This flexibility and versatility of LPS recognition is supplemented by evidence of an extended recognition surface that interacts with the LPS inner core oligosaccharide. The Kdo structure itself provides the first direct confirmation that mild hydrolysis removal of the lipid A from intact LPS exhibiting 4-KdoI substituted KdoII is accompanied by the elimination of KdoII from KdoI C4 [44,45] in a manner entirely equivalent to the β-elimination of phosphate from KdoI C4 through mild acid hydrolysis [21]. This is of some significance in the interpretation of mass spectrometry studies of bacterial LPS structure.

In all subunits where the carbohydrate binding site is openly accessible in the crystal (subunits B and C, both structures), HepI is coordinated to calcium Ca1 as reported for the *H*. *influenzae* Eagan 4A –rfhSP-D structure [21] with KdoI extending between the two binding-site flanking residues, Asp325 and Arg343, both of which interact directly with the bound HepI-Kdo (anhydro) unit (Fig 5). Superposition of the anhydro Kdo furanose rings together with HepI from all these subunits reveals an essentially conserved position of both rings resulting in very similar Asp325 –anhydro Kdo O6 interactions across the structures (2.56–2.80 Å), but significant variations in the Arg343 –Kdo interactions (2.87–3.26 Å) (Table 2). This suggests an important flexibility inherent in the Arg343 –Kdo interaction. Combined with the well documented ability of Arg343 to respond to different ligands through conformational change in the Arg343 side chain [15], this reinforces the importance of the Arg343—ligand interaction as a flexible determinant of specificity.

The R5 bound structure (Fig 6) reveals for the first time that hSP-D has the flexibility and versatility to recognise and bind alternative LPS epitopes if the HepI-Kdo core motif is not accessible or, in the physiological context, is shielded by extended oligosaccharides in the outer core and beyond [21]. In subunit A access to the carbohydrate binding site is restricted with the crystal packing precluding binding of either the R5 or R7 oligosaccharide via the HepI-Kdo pair. The crystal packing does however allow Ca1 binding of the terminal glucose of the longer R5 oligosaccharide, which binds to the SP-D subunit A binding pocket with the oligosaccharide ligand (Fig 6) oriented in a direction roughly orthogonal to the ligand when HepI is bound to Ca1 (Fig 7) as in subunits B and C of both structures (Fig 5). Interestingly, despite the completely different position of HepI with respect to the protein structure, Arg343 remains fundamental to the recognition of the LPS, stabilising the interaction through an Arg343-HepI hydrogen bond (Fig 6 and Table 2) and perhaps contributing to directing the glucose into the binding site [15,16]. While this novel structural demonstration that hSP-D can adopt alternative binding mechanisms for the same LPS is a fortuitous artefact of crystallisation, it clearly shows not only alternative recognition strategies for the same LPS but also the ability to recognise more than just the inner core heptose and Kdo residues in natural LPS ligands. This potentially provides an explanation for the extraordinary ability of hSP-D to recognise such a broad range of gram-negative bacteria by utilising Ca1-dependent binding of not just inner core HepI but also of multiple types of saccharide residue in the LPS [13,21]. The extent of the LPS, and the variety of different carbohydrate residues displayed on bacterial surfaces, provides the opportunity for versatility of recognition by the carbohydrate recognition domain.

**Fig 8:**
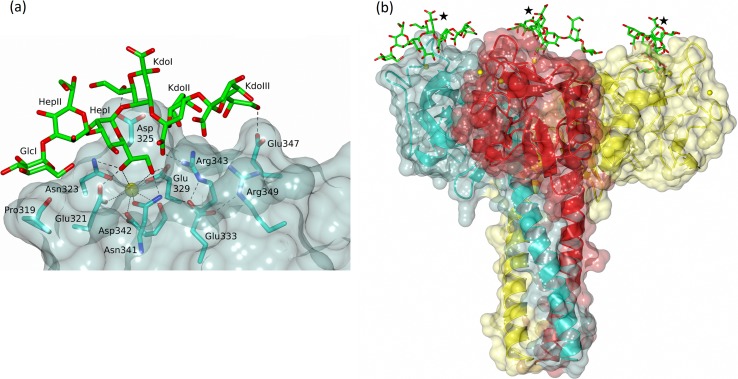
Model of complete *S*. *enterica* R5 oligosaccharide bound to rfhSP-D with the inner core HepI in the Ca1 binding site. KdoI (intact) is positioned to interact with Asp325, with KdoII and KdoIII interacting with Arg343 and Glu347 respectively. (a) subunit C, R5-bound structure (b) the rfhSP-D trimer with R5 similarly placed in each subunit and the link to lipid A from KdoI O2' indicated by ★.

The full *S*. *enterica* sv. Minnesota R5 and R7 LPS oligosaccharides (Fig 1) are known to be recognised by hSP-D [19]. Positioning the full R5 oligosaccharide, including KdoII, KdoIII and a full 6-membered KdoI ring, in the R5-bound structure (subunit C) by overlaying HepI reveals that KdoI may be oriented such that the dihydroxyethyl side chain (7'OH, 8'OH) off C6 interacts either with Asp325 or Arg343. In the orientation shown in Fig 8, KdoII sits between KdoI and the conformationally flexible side chain of Arg343 with both KdoII and KdoIII proximal to the SP-D conserved Glu347 and the SP-A, SP-D conserved Arg349 and Glu333. KdoI O7' interacts with Asp325, KdoII O1' with Arg343, and KdoIII O8' with Glu347. The lipid A linked to KdoI O2' extends out from the CRD and the trimer overall. *Salmonella enterica* mutants with more extended cores and which are recognised by SP-D [19] can also be accommodated in this orientation. For the Rb3 mutant for example (Fig 1), the sugars linked to HepII and Glc1 all extend away from the CRD. This strongly suggests an extended inner core binding site with recognition of the Hep-Kdo motif accompanied by additional binding of inner cores with multiple Kdo structures such as those of *Pseudomonas aeruginosa*, *Escherichia coli and Klebsiella pneumoniae* which are known to be recognised by rfhSP-D [46].

Previous studies of the hSP-D/LPS interaction have shown the importance of Asp325 and Arg343 as oligosaccharide ligand selection mediators, flanking the binding site and directing calcium recognition [13–17], alongside the targeting of the LPS proximal inner core. The structures presented here combine and extend these key elements, revealing the preferred inner core Hep-Kdo binding mechanism alongside a flexibility to recognise alternative epitopes, to provide unique insights into the recognition and binding of a wide variety of gram negative bacteria by hSP-D.
